# Reliability and Validity of Running Cadence and Stance Time Derived from Instrumented Wireless Earbuds

**DOI:** 10.3390/s21237995

**Published:** 2021-11-30

**Authors:** Anouk Nijs, Peter J. Beek, Melvyn Roerdink

**Affiliations:** Faculty of Behavioural and Movement Sciences, Vrije Universiteit Amsterdam, Amsterdam Movement Sciences, van der Boechorststraat 7-9, 1081 BT Amsterdam, The Netherlands

**Keywords:** cadence, stance time, accelerometer, agreement

## Abstract

Instrumented earbuds equipped with accelerometers were developed in response to limitations of currently used running wearables regarding sensor location and feedback delivery. The aim of this study was to assess test–retest reliability, face validity and concurrent validity for cadence and stance time in running. Participants wore an instrumented earbud (new method) while running on a treadmill with embedded force-plates (well-established method). They ran at a range of running speeds and performed several instructed head movements while running at a comfortable speed. Cadence and stance time were derived from raw earbud and force-plate data and compared within and between both methods using *t*-tests, *ICC* and Bland–Altman analysis. Test–retest reliability was good-to-excellent for both methods. Face validity was demonstrated for both methods, with cadence and stance time varying with speed in to-be-expected directions. Between-methods agreement for cadence was excellent for all speeds and instructed head movements. For stance time, agreement was good-to-excellent for all conditions, except while running at 13 km/h and shaking the head. Overall, the measurement of cadence and stance time using an accelerometer embedded in a wireless earbud showed good test–retest reliability, face validity and concurrent validity, indicating that instrumented earbuds may provide a promising alternative to currently used wearable systems.

## 1. Introduction

Using an instrumented treadmill to measure gait parameters in the lab is a widely accepted and commonly applied method [1,2]. The force platforms embedded in the treadmill allow accurate recording of all steps in a trial, which constitutes a significant improvement over the previously used force platforms embedded in the floor that could only measure a single stance [1]. However, measurements with instrumented treadmills are restricted to the lab and, although biomechanically similar [3], some aspects of treadmill gait differ from overground gait because of the often fixed imposed speed in combination with the limited treadmill surface [4,5,6]. Moreover, the option of providing real-time feedback for long-term performance improvement is limited in a lab as it requires that participants regularly return to the lab. Various methods for gait analysis using inertial sensors have been developed for both walking and running. These inertial sensors can measure multiple steps, and are mobile, allowing measurement and feedback in the field [7,8,9,10,11,12,13,14].

Although the use of inertial sensors is popular among runners, usually in the form of *wearables* [15,16,17], they still have limitations that need to be resolved. First, the wearable sensors are often located on the legs [7,8,10,13] or the lower back [9,13], which is not very practical for the runner. In addition, the sensor placement can influence output variables and there is still no consensus on the optimal location for sensor placement [11,12,13,18,19,20], although algorithms for the determination of cadence have been developed that do not depend on sensor location [21]. Second, real-time feedback of gait parameters such as cadence and contact times derived from inertial sensors is often provided visually on a watch, looking at which interferes with running [15,22]. Auditory instructions or feedback on gait parameters, for example through auditory pacing [23] or sonification [24,25], is therefore preferable [26]. Third, previous research indicated that runners dislike carrying their phone and would prefer using an all-in-one device [16]. Thus, although many wearable sensors have been developed, there are still improvements to be made regarding the location on the body and feedback delivery.

Ear-based sensors could provide a solution to these limitations. The location of an ear-based sensor solves the placement problem associated with wearables, and neither the sensor itself nor the provision and pickup of feedback will interfere with running. In addition, the sensor may be influenced less by vibrations due to the soft tissue between sensor and bone [27], since it is positioned close to the skull. After all, accelerations are highest in the lower extremities and decrease along the vertical axis of the body, with the lowest accelerations being measured on the head [28]. Still, previous research has shown that an ear-based accelerometer could be used to distinguish between medium to high level activity [20], between impaired and non-impaired gait [29], and between walking and running [30]. An ear-based accelerometer can also be used to measure parameters related to the gait cycle and force loading in walking [31,32,33,34], especially at faster walking speeds, during which accelerations are higher [32]. It was suggested that the larger difference between the ear-based accelerometer-derived parameters and the treadmill-derived parameters when walking at lower speeds was due to more incidental head movements, such as nodding and looking around, which caused measurement artefacts [32].

Instrumented wireless earbuds, equipped with embedded accelerometers to measure and process acceleration data, were developed by Dopple B.V. (Assen, The Netherlands). These earbuds can provide real-time auditory feedback of gait parameters during the run, essentially creating an all-in-one device. The main purpose of the present study was to assess the test–retest reliability, face validity and concurrent validity of running gait parameters derived from accelerometer data collected with the instrumented earbuds of Dopple B.V., and thus, to extend the findings regarding the use of ear-worn sensors in walking [31,32,33,34] to running. We focused on cadence and stance time, as recent literature proposed that the combination of these gait parameters can be used to describe running technique [35]. Cadence and stance time were derived from concurrent earbud and force-plate data for a range of running speeds. We assessed their test–retest reliability for both measurement methods, their face validity in terms of changes in to-be-expected directions with increasing running speed (i.e., higher cadence and shorter stance times [35]), and concurrent validity in terms of their agreement between both measurement methods. In addition, we looked at the between-methods agreement of the derived gait parameters when participants performed incidental head movements upon instruction while running at a comfortable running speed.

## 2. Materials and Methods

Fourteen healthy runners (6 male/8 female), 36 ± 13 years of age (mean ± standard deviation; *M* ± *SD*) with body weights of 71.4 ± 14.1 kg, participated in the study. All participants provided written informed consent before participating. The protocol was in accordance with the Declaration of Helsinki and approved by the Scientific and Ethical Review Board of the Faculty of Behavioural and Movement Sciences of the Vrije Universiteit Amsterdam (VCWE-2019-006R1).

**Figure 1:**
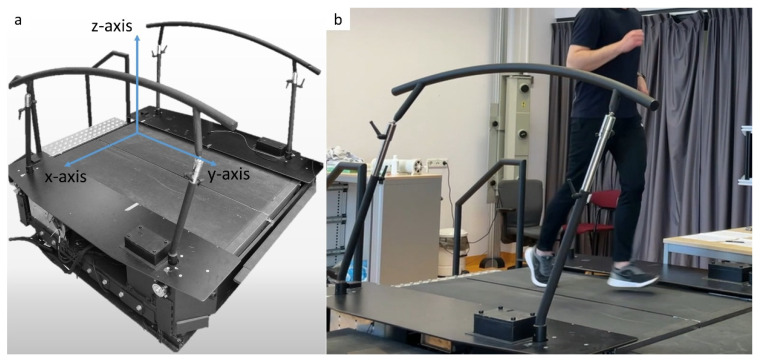
Instrumented treadmill. (**a**) The instrumented treadmill and the coordinate system of the embedded force plates. (**b**) The instrumented treadmill during a measurement.

**Figure 2:**
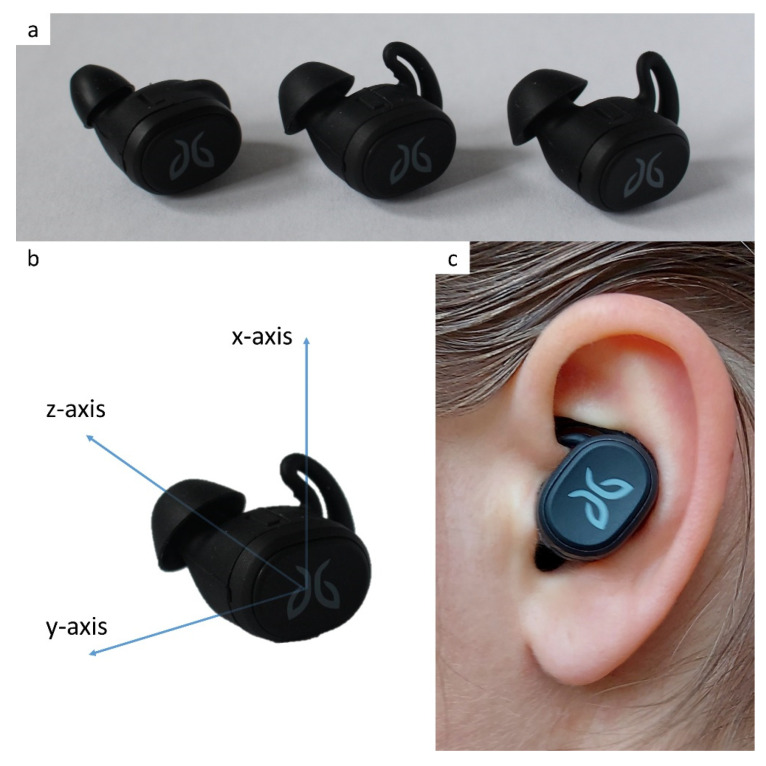
Instrumented wireless earbud. (**a**) Three earbuds with differently sized attachments. (**b**) The coordinate system of the accelerometer in the earbud. (**c**) The snugly fit earbud in the ear.

Participants ran on an instrumented treadmill (Motek, Amsterdam), which was equipped with force plates that measured the 3D ground reaction force (Figure 1). Participants wore an earbud equipped with a 3D accelerometer (LIS2DW12, STMicroelectronics, Geneva, Switzerland) in their left ear (Figure 2). The earbud came with three attachments in different sizes, to ensure a snug fit for every participant (Figure 2a). Data were sampled at 500 Hz in the force plate and at 800 Hz in the accelerometer.

### 2.1. Data Collection

Each participant completed all conditions in a repeated-measures design. Before the measurements began, participants ran on the treadmill at a comfortable speed to warm-up and familiarize themselves with running on the treadmill. Then, the maximum running speed was determined by discussing it with the participant and optionally trying to run at that speed for a short while. Participants were told that they could abort a trial at any time if the speed turned out to be too high.

The participants were invited to run at four speeds ranging from 7 km/h, around which the transition from walking to running occurs [36], to 16 km/h, which was the maximum speed of the belt, with increments of 3 km/h, i.e., they ran at 7 km/h, 10 km/h, 13 km/h, and the maximum running speed of the participant, up to a maximum of 16 km/h. Each speed was measured for one minute. During the speed trials, no instruction regarding head movement was given. The order in which participants performed the speed conditions was randomized. After having completed all speed conditions once, participants performed a trial with instructed head movements while running at a comfortable running speed. During this head-movement trial, participants started running while keeping the head in a neutral position (facing forward). After about 15 s, participants were verbally instructed by the researcher to perform a particular head-movement condition every 15 s. Participants received seven such instructions, pertaining to a variety of different head-movement conditions (Table 1), which were explained beforehand. They were instructed to perform the head movements as realistically as they could during a run. The order in which those head-movement conditions were performed within the head-movement trial was random. After this trial with instructed head movements, the participants performed all speed conditions a second time in the same order to establish the test–retest reliability.

Each trial started with a jump on the instrumented treadmill for synchronization purposes. Subsequently, the treadmill was started and brought up to speed. At the end of each trial, the speed of the treadmill was decreased to standstill. To avoid fatigue, participants were allowed a break in between trials for as long as they deemed necessary to recover. The experiment was concluded when the participants had completed all trials.

### 2.2. Data Analysis

All calculations were conducted in MATLAB (MathWorks^®^, R2018b). The earbud data were resampled to 500 Hz and synchronized to the force-plate data manually by overlapping the jumps in the different datasets. Differences in maximum running speed and technical problems led to an incomplete dataset, within which not all speeds could be compared for all participants. Therefore, the sample size was reported for each comparison, and the maximum speed condition was not analyzed statistically due to the small sample size.

For all trials, the time instances of foot strike (t_foot strike_) and foot off (t_foot off_) were determined based on the force data and the acceleration data from the earbud. For the different speed conditions, data for 50 s of consistent running were used for the analysis, and for the head-movement conditions, data for 10 s around the head movement were used.

**Figure 3:**
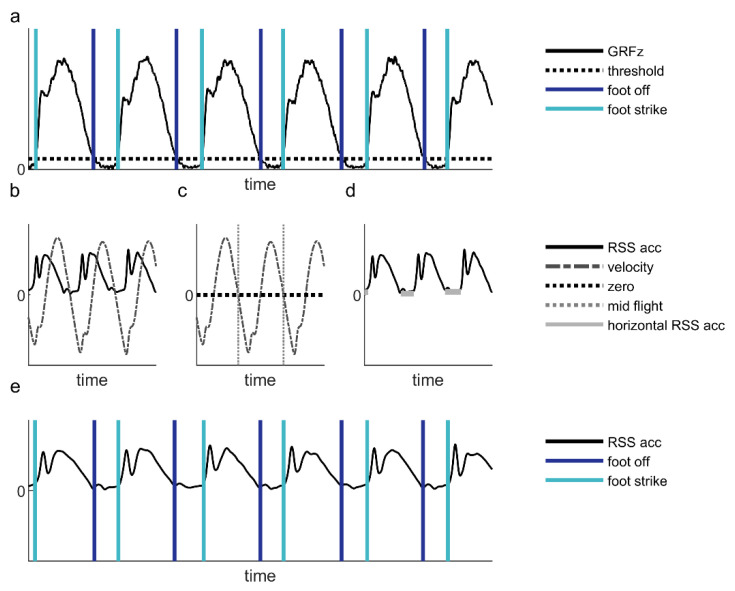
Example of representative data and an overview of the event detection. (**a**) The vertical ground reaction force (GRFz), the threshold (horizontal dotted line) and the instants of foot off and foot strike (vertical blue lines). (**b**) The root sum square of the acceleration data (RSS acc, solid black line) and the velocity (dashed grey line) during one second of running. (**c**) The velocity and its negative zero crossings (mid-flight, grey dotted vertical lines). (**d**) The root sum square of the acceleration data and the width of the horizontal sections (grey horizontal lines) around each mid-flight. (**e**) The root sum square of the acceleration data (RSS acc, solid black line) during two seconds of running. The vertical blue lines indicate instants of foot off and foot strike.

Force plate. Foot-strike and foot-off events were determined as the time instances at which the vertical component of the ground reaction force crossed a threshold of 25% of body weight in the upward and downward direction, respectively (Figure 3a). Based on the determined events, cadence (in steps/min) was calculated as:

(1) $$\mathrm{Cadence}=\frac{1}{n}\overset{n}{\underset{i=2}{\sum }}(\frac{60}{t_{\mathrm{foot}\;\mathrm{strike}\;\left(i\right)}-t_{\mathrm{foot}\;\mathrm{strike}\left(i-1\right)}})$$

and stance time (in s) was calculated as:

(2) $$\mathrm{Stance}\;\mathrm{time}=\frac{1}{n}\sum_{i=1}^{n}(t_{\mathrm{foot}\;\mathrm{off}\left(i\right)}-t_{\mathrm{foot}\;\mathrm{strike}\left(i\right)}),$$

where *n* is the number of steps and t_foot strike(i+1)_ > t_foot off(i)_ > t_foot strike(i)_.

Earbud. A custom-written algorithm, which was developed specifically for the instrumented earbud by Dopple B.V., was used to determine cadence and stance time. This algorithm comprises a sliding window analysis (2 s, 0.6 s overlap) for real-time gait-event estimation. For each sample in a window, it first calculates the root sum square of the 3D acceleration to limit the effect of orientation differences. Then, the integral over time of this root sum square series is calculated to represent the velocity time series (Figure 3b). Next, the negative zero crossings of the velocity are determined, which roughly represent the middle of the flight phase (Figure 3c). Finally, for every flight phase identified in the window, the corresponding width of the horizontal section in the root sum square of the acceleration is determined, whose boundaries provided estimates of foot-off and foot-strike events (Figure 3d,e). All events were collated and duplicates were removed before calculating the cadence and stance time according to Equations (1) and 2, respectively.

### 2.3. Statistical Analysis

The statistical analysis was performed in IBM SPSS Statistics 25. Results were deemed significant when *α* < 0.05.

For each speed, within-method test–retest reliability was assessed for cadence and stance time using a two-tailed paired-samples *t*-test to identify systematic biases between test and retest, accompanied by the intra-class correlation coefficient (*ICC*) based on a single-rater, absolute agreement, 2-way mixed-effects model (values <0.5, 0.5 to 0.75, 0.75 to 0.9, and >0.9 indicate poor, moderate, good, and excellent agreement, respectively [37]). Bland–Altman analysis was used to establish the bias and 95% limits of agreement [38]. Bland–Altman plots were made to visualize the bias and limits of agreement.

Face validity was determined per method by comparing cadence and stance time over speeds with a one-way repeated-measures ANOVA, with paired-samples *t*-tests for post-hoc comparisons. Greenhouse–Geisser corrections were applied when Mauchly’s test of sphericity was significant.

The between-methods agreement for cadences and stance times was assessed for the test and retest separately, as well as for the two tests combined [39], again using paired-samples *t*-tests accompanied by *ICC* and Bland–Altman analysis.

## 3. Results

**Figure 4:**
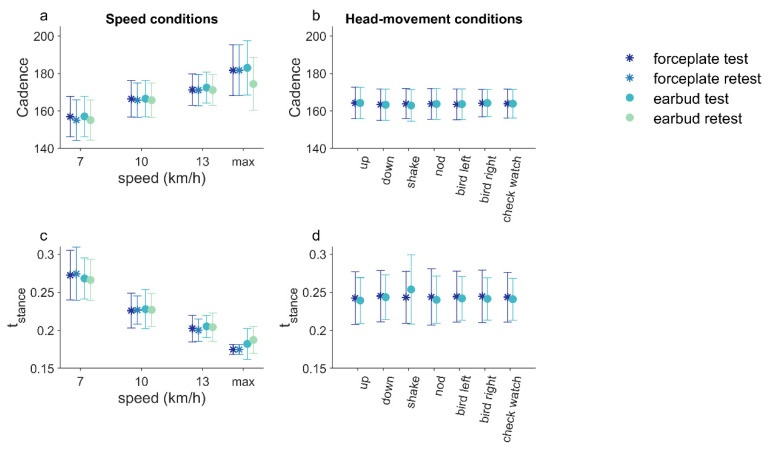
Mean and standard deviation of cadence (**a**,**b**) and stance time (**c**,**d**) for the two methods for test and retest for the different speed conditions (**a**,**c**) and for the head-movement conditions (**b**,**d**).

Datafiles of the force and acceleration data for all trials per participant, together with the calculated variables, can be found in the Supplementary Materials. The mean cadence and stance time, as measured for the different speeds by the two methods in test and retest, are shown in Figure 4.

### 3.1. Within-Method Reliability: Speed Conditions

**Figure 5:**
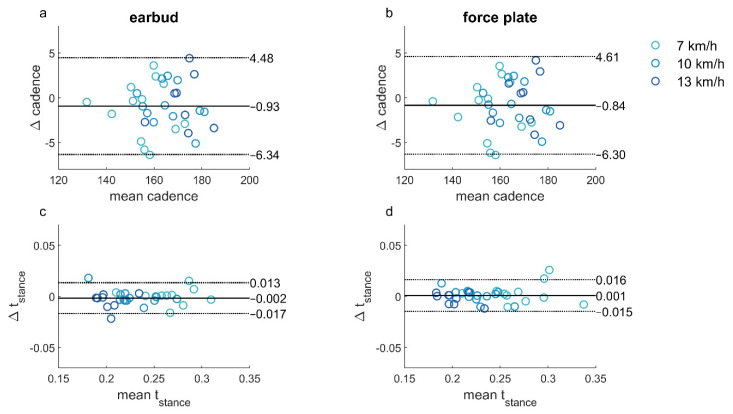
Bland–Altman plots comparing the test and retest data for the earbud (**a**,**c**) and the force-plate (**b**,**d**). Each dot represents the test–retest combination for one participant at a particular speed.

Test–retest biases were not significant for both methods (Figure 5; Table 2). The *ICC* showed good-to-excellent agreement for the earbud method (Table 2) and excellent agreement for the force-plate method (Table 2). The Bland–Altman plots showed similar limits of agreement for both methods and indicated no dependence of the difference on the mean (Figure 5).

### 3.2. Face Validity: Difference over Speed Conditions in Expected Direction

For both methods, cadence increased significantly with speed (Figure 4a; earbud: *F*(1.04, 8.35) = 11.16, *p* = 0.009; force plate: *F*(1.04, 8.34) = 10.97, *p* = 0.01), with significant post-hoc differences between 13 km/h and the other two speed conditions (*p* < 0.017), but no significant difference between 7 and 10 km/h, for both the earbud and force-plate methods (*p* = 0.128; *p* = 0.126, respectively). For both methods, stance time decreased significantly with increasing speed (Figure 4c; earbud: *F*(2, 12) = 110.74, *p* = 0.000; force plate: *F*(1.12, 6.72) = 116.93, *p* = 0.000), with significant post-hoc differences between the three speed conditions (*p* < 0.001).

### 3.3. Between-Methods Agreement: Speed Conditions

**Figure 6:**
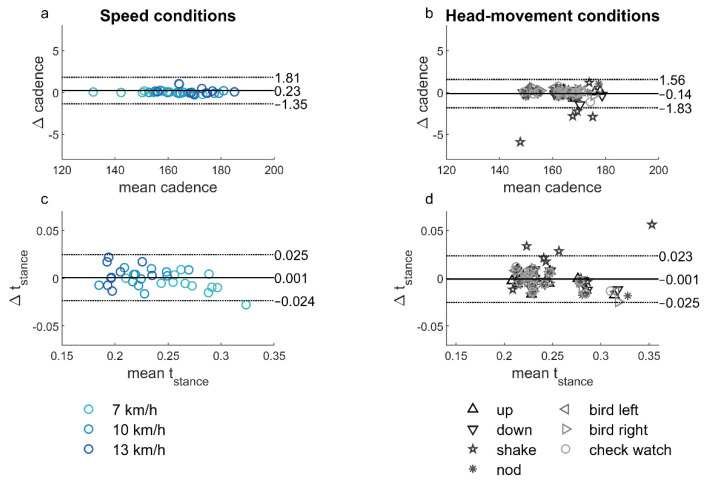
Bland–Altman plots showing the agreement of cadence (**a**,**b**) and stance time (**c**,**d**) between earbud and force-plate methods for the different speed conditions (test and retest combined; (**a**,**c**)) and the head-movement conditions (**b**,**d**).

For most speeds, no significant between-methods biases in cadence and stance time were found (Figure 6a,c; Table 3). Only the stance time in the retest at 7 km/h had a significant bias of −0.008 ± 0.010 s (*p =* 0.02; Table 3), showing that the average stance time as measured with the force plate was 0.008 s longer than the average stance time as measured with the earbud. The test, retest and combined *ICC* showed excellent agreement for the cadence for all speeds (Table 3) and for the stance time at 7 and 10 km/h (Table 3); the stance time at 13 km/h showed moderate agreement when test and retest were combined, with poor agreement on the test and good agreement on the retest (Table 3). Bland–Altman plots revealed no clear dependence of the difference on the mean (Figure 6a,c).

### 3.4. Between Methods Agreement: Instructed Head-Movement Conditions

The between-methods bias was not significant for both cadence and stance time for all head movements (Table 4; Figure 6b,d). The *ICC* showed excellent agreement for cadence for all head movements (Table 4), and for stance time for all head movements except shaking the head, for which the agreement was good (Table 4). The Bland–Altman plots also revealed that the difference between the methods was largest for the instruction to shake the head (Figure 6d).

## 4. Discussion

In this study, we determined cadence and stance time from accelerometer data collected in an earbud (new method) and force-plate data (well-established method) for a range of running speeds. For both methods, we assessed the test–retest reliability per speed condition and face validity over speed conditions (i.e., does cadence increase and stance time decrease with increasing speed?). In addition, concurrent validity was assessed by determining the between-methods agreement of cadence and stance time for the different speeds, as well as for a variety of instructed head-movements performed at a comfortable running speed. The test–retest reliability was good to excellent for both earbud and force-plate methods for both cadence and stance time, with similar limits of agreement. Face validity was also good—with significant differences in cadence and stance time with running speed in to-be-expected directions [35]—for both methods. We found excellent between-methods agreement for the cadence determined over the range of speeds and during the instructed head movements. The between-methods agreement for the stance time was good-to-excellent, with the agreement being less good for 13 km/h and while shaking the head. For the retest at 7 km/h, the between-methods bias in stance time was significant, although small (8 ms), while the corresponding agreement was excellent (Table 3).

Although both test–retest reliability and between-methods agreement were excellent, the limits of agreement for cadence for between-methods differences were considerably smaller than for test–retest differences within the methods (about three times; compare Figure 6a with Figure 5a,b, respectively). Variations found in test–retest reliability can partly be attributed to the variation in the performance of the participant [40], especially given the similarity in the limits of agreement and *ICC* for the test and retest assessments of the earbud and force-plate methods (Figure 5a,b). These results indicate that the two methods can be used interchangeably to measure running cadence over a range of speeds.

For the stance time, the limits of agreement were not smaller for the between-methods agreement compared to the test–retest reliability, although the limits of agreement on the test–retest comparisons were still similar for the earbud and force-plate methods (Figure 5c,d). It should be noted that the stance time, as calculated from the force-plate data, is dependent on the chosen threshold. Different thresholds are used in the literature (e.g., set thresholds [1,12,13,41], weight-dependent thresholds [39], or variability-dependent thresholds [2]). A higher force-plate threshold leads to systematically shorter stance times and vice versa, which would influence the bias between the force-plate and earbud data. In this study, we chose to use a weight-dependent threshold in view of the large weight differences between participants, which could have influenced event detection when using a set threshold. Future research should further examine the effects of threshold choice in force plate-based gait analysis and, ideally, culminate in evidence-based guidelines.

**Figure 7:**
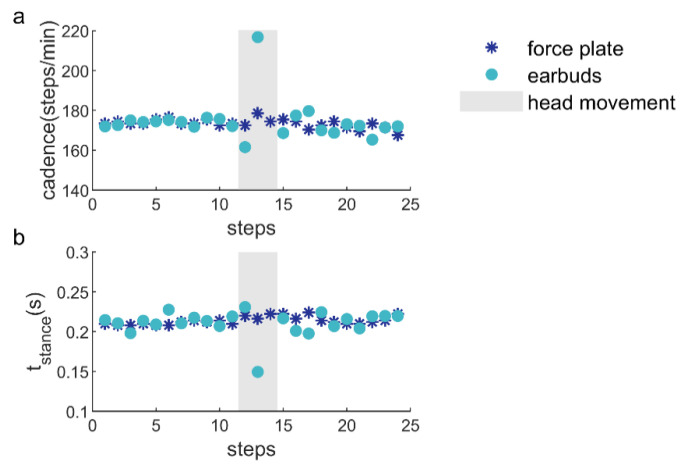
Example of a representative part of the instructed head-movement trial for a single participant for the head-movement condition ‘shake’, with step-based estimates of cadence (**a**) and stance time (**b**), over which the means were calculated. The shaded area represents steps with head shakes.

Overall, the between-methods comparisons yielded similar agreements for the different speeds and the different instructed head movements, indicating that incidental head movements did not have a substantive impact on the derived cadences and stance times. For this study, we calculated the mean cadence/stance time over a certain time window. For the instructed head movements, this meant the mean cadence/stance time for a window of 10 s around the head movement. Some of the instructed head movements, in particular the head shake, tended to cause errors in the earbud-based cadence/stance time estimates for the steps during which the particular head movement was performed, but not for the other steps (Figure 7). The size of the window over which the mean is taken will influence the impact of errors in cadence/stance time associated with incidental head movements. If this is found to be a problem, using measures that are less sensitive to outliers, such as the median, will further limit the impact of incidental head movements on calculated variables.

The relative differences found in cadence and stance time between the two methods are similar to the relative differences found in some previous studies that validated the use of ear-worn sensors in walking [31,34], and smaller than in other studies [32]. Our results extend the finding that ear-worn sensors can be used for gait analysis during walking to running, thus broadening the applicability of this type of sensors.

In conclusion, our results showed that cadence and stance time during running can be derived reliably from instrumented wireless earbuds equipped with an accelerometer, with good face validity and concurrent validity. Combined with the previously mentioned advantage of having a practical sensor location and the possibility to provide on-line auditory instructions or feedback, such as pacing to modulate cadence [23] and impact forces [42], instrumented earbuds may become a promising alternative to currently used wearable systems and the earbuds could even be developed into a promising all-in-one feedback device for both treadmill and outdoor running. A follow-up study comparing cadence and stance times derived using the earbud method to results obtained via a different validated method that is suitable for deriving cadence and stance times outdoors is recommended to substantiate this promise. Aspects of running outdoors that could be considered are speed variability and higher inter-step variability due to turns, uneven terrain and obstacles, and impact differences due to surface characteristics [43].

## Figures and Tables

**Table 1:** Head-movement conditions, performed in random order in a single trial.

Instructed Head Movements	Explanation of the Instructions
Look up	Rotate the head upwards to look toward the ceiling for a second
Look down	Rotate the head downwards to look toward the floor for a second
Shake your head	Rotate the head sideways to both sides quickly for a few repetitions
Nod	Rotate the head vertically up and down quickly for a few repetitions
Look at a bird flying by on the left	Rotate the head diagonally in a slight upward direction to the left for a second
Look at a bird flying by on the right	Rotate the head diagonally in a slight upward direction to the right for a second
Look at the time/check your watch (wrist)	Move your preferred arm in front of your body and look down towards your wrist, as if you were to check the time on a watch

**Table 2:** Means, standard deviations and test–retest reliability of the cadence and stance time as measured with the earbud and force-plate methods.

	Speed (km/h)	Test		Retest						
		*M*	*SD*	*M*	*SD*	*bias*	*SD*	*t*(*df*)	*p*	*ICC*
Earbuds										
Cadence	7	156.50	11.01	155.15	10.74	−1.35	3.19	*t*(12) = −1.53	0.15	0.953
(steps/min)	10	166.55	9.72	165.77	9.10	−0.78	2.22	*t*(11) = −1.22	0.25	0.971
	13	171.55	8.29	171.21	8.31	−0.49	3.01	*t*(7) = −0.46	0.66	0.946
	max	175.86	14.83	174.44	13.97	−1.42	3.38	N = 3		
Stance time	7	0.266	0.027	0.266	0.027	0.000	0.008	*t*(10) = 0.02	0.99	0.960
(s)	10	0.228	0.026	0.227	0.022	−0.001	0.007	*t*(11) = −0.48	0.64	0.960
	13	0.205	0.015	0.200	0.015	−0.005	0.008	*t*(7) = −1.73	0.13	0.817
	max	0.187	0.022	0.188	0.018	0.001	0.006	N = 3		
Force plate										
Cadence	7	156.47	11.01	155.13	10.83	−1.34	3.27	*t*(12) = −1.48	0.17	0.951
(steps/min)	10	166.54	9.76	165.80	9.14	−0.74	2.20	*t*(11) = −1.17	0.27	0.972
	13	171.39	8.38	171.13	8.34	−0.26	2.91	*t*(8) = −0.27	0.79	0.945
	max	180.08	14.96	178.98	14.62	−1.10	2.83	N = 4		
Stance time	7	0.271	0.034	0.274	0.035	0.003	0.011	*t*(10) = 1.00	0.34	0.953
(s)	10	0.226	0.023	0.227	0.019	0.001	0.006	*t*(11) = 0.45	0.67	0.956
	13	0.203	0.017	0.200	0.015	−0.002	0.006	*t*(8) = −1.13	0.29	0.938
	max	0.175	0.007	0.177	0.008	0.002	0.005	N = 4		

**Table 3:** Between-methods bias and agreement of cadence and stance time between earbud and force-plate methods for the speed conditions (bias = earbud − force plate).

	Speed (km/h)		Force plate		Earbud						
			*M*	*SD*	*M*	*SD*	*bias*	*SD*	*t(df)*	*p*	*ICC*
Cadence (steps/min)	7	Test	156.47	11.01	156.50	11.01	0.10	0.28	*t*(13) = 1.32	0.21	1.000
		Retest	155.13	10.83	155.15	10.74	0.02	0.17	*t*(12) = 0.53	0.61	1.000
		Combined	156.37	10.59	156.47	10.61	0.09	0.27	*t*(13) = 1.33	0.21	1.000
	10	Test	166.54	9.76	166.55	9.72	0.02	0.18	*t*(11) = 0.32	0.76	1.000
		Retest	165.80	9.14	165.77	9.10	−0.02	0.14	*t*(11) = −0.59	0.57	1.000
		Combined	166.17	9.39	166.16	9.35	−0.00	0.14	*t*(11) = −0.09	0.93	1.000
	13	Test	171.39	8.38	172.52	8.30	0.05	0.21	*t*(7) = 0.63	0.55	1.000
		Retest	171.13	8.34	172.03	8.48	0.08	0.27	*t*(8) = 0.84	0.43	0.999
		Combined	171.26	8.23	171.42	8.13	0.16	0.38	*t*(8) = 1.27	0.24	0.999
	max	Test	180.08	14.96	175.86	14.83	1.28	1.99	N = 5		
		Retest	178.98	14.62	174.44	13.97	0.07	0.15	N = 3		
		Combined	181.33	13.37	182.63	14.39	1.29	2.01	N = 5		
Stance time (s)	7	Test	0.273	0.033	0.268	0.027	−0.004	0.011	*t*(11) = −1.40	0.19	0.933
		Retest	0.274	0.035	0.266	0.027	−0.008	0.010	*t*(10) = −2.77	0.02	0.924
		Combined	0.274	0.033	0.268	0.027	−0.006	0.010	*t*(11) = −2.02	0.07	0.933
	10	Test	0.226	0.023	0.228	0.026	0.002	0.009	*t*(11) = 0.73	0.48	0.940
		Retest	0.227	0.019	0.227	0.022	0.000	0.009	*t*(11) = 0.01	0.99	0.916
		Combined	0.227	0.021	0.227	0.024	0.001	0.008	*t*(11) = −0.48	0.71	0.934
	13	Test	0.198	0.011	0.205	0.015	0.007	0.016	*t*(7) = 1.29	0.24	0.212
		Retest	0.200	0.015	0.204	0.019	0.004	0.010	*t*(8) = 1.22	0.26	0.825
		Combined	0.201	0.016	0.207	0.017	0.005	0.012	*t*(8) = 1.29	0.23	0.732
	max	Test	0.175	0.006	0.182	0.020	0.007	0.022	N = 5		
		Retest	0.181	0.002	0.188	0.017	0.007	0.020	N = 3		
		Combined	0.176	0.006	0.182	0.019	0.007	0.021	N = 5		

**Table 4:** Agreement of the cadence and stance time between the force-plate and earbud methods for the head-movement conditions.

	Head Movements	Force Plate		Earbud						
		*M*	*SD*	*M*	*SD*	*bias*	*SD*	*t*(*df*)	*p*	*ICC*
Cadence	Up	164.32	8.45	164.31	8.32	−0.01	0.34	*t*(12) = −0.07	0.94	0.999
	Down	163.43	8.39	163.33	8.28	−0.09	0.47	*t*(12) = −0.71	0.49	0.998
	Shake	163.92	8.04	162.94	8.43	−0.98	1.96	*t*(12) = −1.80	0.10	0.967
	Nod	163.74	8.19	163.76	8.32	0.02	0.42	*t*(12) = 0.17	0.87	0.999
	Bird left	163.60	8.19	163.70	8.20	0.09	0.27	*t*(12) = 1.31	0.22	0.999
	Bird right	164.21	7.35	164.26	7.19	0.05	0.31	*t*(12) = 0.58	0.58	0.999
	Check watch	163.94	7.70	163.91	7.60	−0.04	0.42	*t*(12) = −0.31	0.76	0.999
Stance time	Up	0.242	0.035	0.239	0.030	−0.003	0.009	*t*(12) = −1.31	0.22	0.962
	Down	0.245	0.034	0.244	0.029	−0.002	0.008	*t*(12) = −0.70	0.50	0.970
	Shake	0.243	0.034	0.254	0.045	0.010	0.020	*t*(12) = 1.86	0.09	0.855
	Nod	0.244	0.037	0.240	0.031	−0.004	0.009	*t*(12) = −1.41	0.18	0.958
	Bird left	0.245	0.034	0.242	0.029	−0.002	0.009	*t*(12) = −1.03	0.32	0.962
	Bird right	0.245	0.034	0.242	0.028	−0.003	0.009	*t*(12) = −1.16	0.27	0.954
	Check watch	0.244	0.033	0.241	0.028	−0.003	0.009	*t*(12) = −1.06	0.31	0.957

## Data Availability

The data presented in this study are available in the Supplementary Materials: Datafiles.

## Supplementary Materials

The data are available online at https://www.mdpi.com/article/10.3390/s21237995/s1.
