# Supplementary material for: Reliability and Validity of Running Cadence and Stance Time Derived from Instrumented Wireless Earbuds
Source: Sensors (Basel). 2021 Nov 30;21(23):7995. doi: 10.3390/s21237995 (PMC8659722; doi:10.3390/s21237995)
Supplement: Supplementary file 1 [file sensors-21-07995-s001.zip › description data.pdf]

October 2021

This dataset belongs to the research article entitled
'Reliability and validity of running cadence and stance time derived from instrumented wireless earbuds'

Any questions regarding the dataset can be addressed to a.nijs@vu.nl

The dataset is organized into csv files containing the 3D force plate data and 3D acceleration data per participant and a csv file containing calculated cadence and stance time per condition per participant.

The files containing the 3D force plate data and 3D acceleration data for each participant are named as follows:

Speeds test	'*Data_P##_speeds_1.csv*'
Speeds retest	'*Data_P##_speeds_2.csv*'
Head movements	'*Data_P##_HeadMovements.csv*',

where ## is the participant number. The data is organized in columns per condition. The first column *'time'* represents the timestamps associated with the corresponding data.

The file containing calculated cadence and stance time is named *'Calculated_variables.csv'*. Each row represents the data for one participant, and each column represents a variable for a condition.

Columns are named according to *'Variable_condition_method'*, for example:

'Cadence_7_1_fp'	Cadence calculated based on the force plate for the first trial (test) at a speed of 7km/h
'StanceTime_max_2_eb'	Stance time calculated based on the second trial (retest) at the max speed
'StanceTime_up_eb'	Stance time calculated based on the earbuds for the head movement 'up'
